# Diagnosis and treatment of breast cancer metastasis to thyroid: a case report and literature review

**DOI:** 10.3389/fsurg.2026.1787683

**Published:** 2026-05-25

**Authors:** TaiXu Jiang, YueNan Zheng, HongShi Shi, Liang He, MoFei Wang

**Affiliations:** 1 Department of Thyroid, Breast and Hernia Surgery, Affiliated Hospital of Inner Mongolia Minzu University, Tongliao, Inner Mongolia Autonomous Region, China; 2 Department of Thyroid Surgery, The First Hospital of China Medical University, Shenyang, Liaoning Province, China

**Keywords:** breast cancer, case report, diagnosis, secondary malignant tumor, thyroid metastatic cancer, treatment

## Abstract

Metastatic thyroid cancer originating from breast cancer is rare and often misdiagnosed in clinical practice. Early identification, diagnosis, and treatment are the main way to improve prognosis. This case reports describes a unique case of breast cancer metastasis to the thyroid 16 years after the diagnosis of breast cancer in order to enhance clinicians awareness to this rare disease.

**Case report:**

A 54-year-old woman was diagnosed with left breast ductal carcinoma + invasive lobular carcinoma (T2N2M0, stage IIIA) 16 years ago. Her condition became stable after radiotherapy, chemotherapy, and endocrine therapy. Recent physical examination indicated a C-TIRADS-4B mass in the right thyroid gland, which was indicative of thyroid malignancy. Radical thyroid surgery was performed for thyroid cancer. Postoperative pathology and immunohistochemistry revealed that the cancer originated from the breast and positron emission tomography–computed tomography (PET-CT) indicated multiple systemic bone metastases. After multidisciplinary consultation, endocrine therapy and chemotherapy were administered. Currently, the patient is alive.

**Conclusion:**

Breast cancer metastasis to the thyroid is rare. If a patient with a history of breast cancer has newly developed thyroid nodules, the clinician should highly suspect the origin of the tumor. Preoperative FNAB and immunohistochemistry are the main methods for diagnosis and surgery is the major treatment.

## Introduction

Thyroid metastatic cancer (TMC) refers to non-primary thyroid tumors metastasized to the thyroid gland, with an incidence of only 0.36% (1). In most cases of TMC, the primary tumors are mainly renal cancer (48.1%), colorectal cancer (10.4%), and lung cancer (8.3%), and only 7.8% originate from breast cancer (2). Because of lack of specific clinical symptoms and characteristics, and a long interval between the diagnosis of the primary malignant tumor and the discovery of thyroid metastasis, it is often misdiagnosed in clinical practice, leading to delayed treatment. Here, we describe a rare case of breast cancer that metastasized to the thyroid gland 16 years after the operation. In this report, we share our diagnosis and treatment process for this disease to strengthen the clinician's understanding of TMC.

## Case report

**Figure 1:**
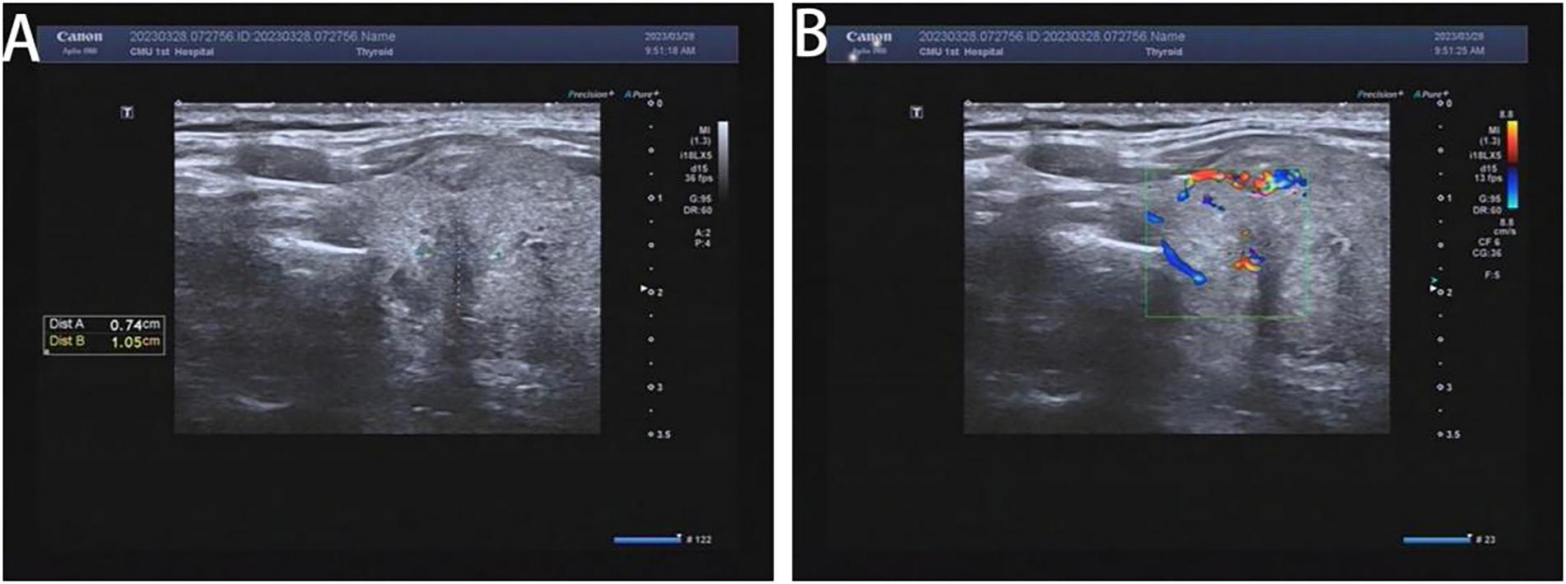
Thyroid ultrasound: **(A)** shows unclear boundaries and irregular shape in the right lobe of the thyroid gland with low echogenicity mass. **(B)** Shows punctate blood flow within the thyroid gland mass.

**Figure 2:**
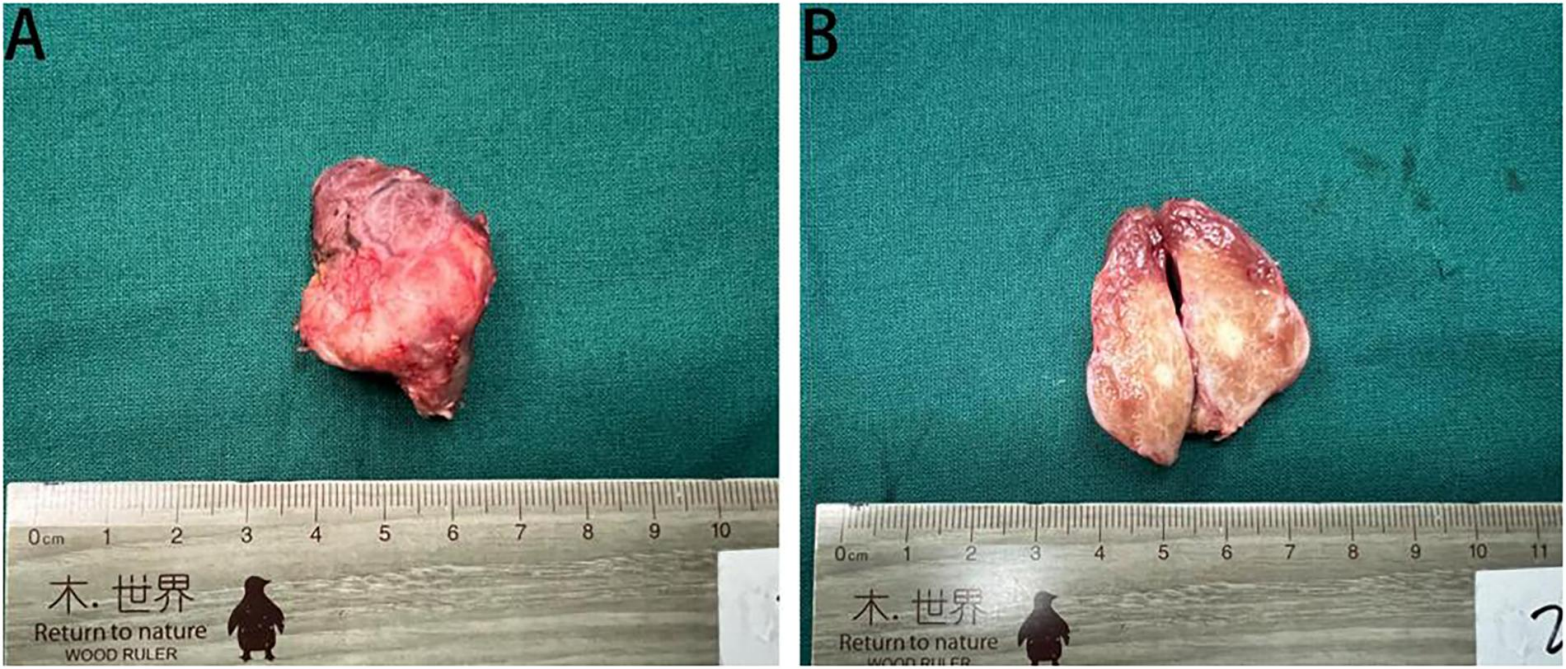
**(A)** Represents the excised right lobe of the thyroid gland. **(B)** Represents the mass in the right lobe of the thyroid gland with a gray-white border and a yellowish center.

**Figure 3:**
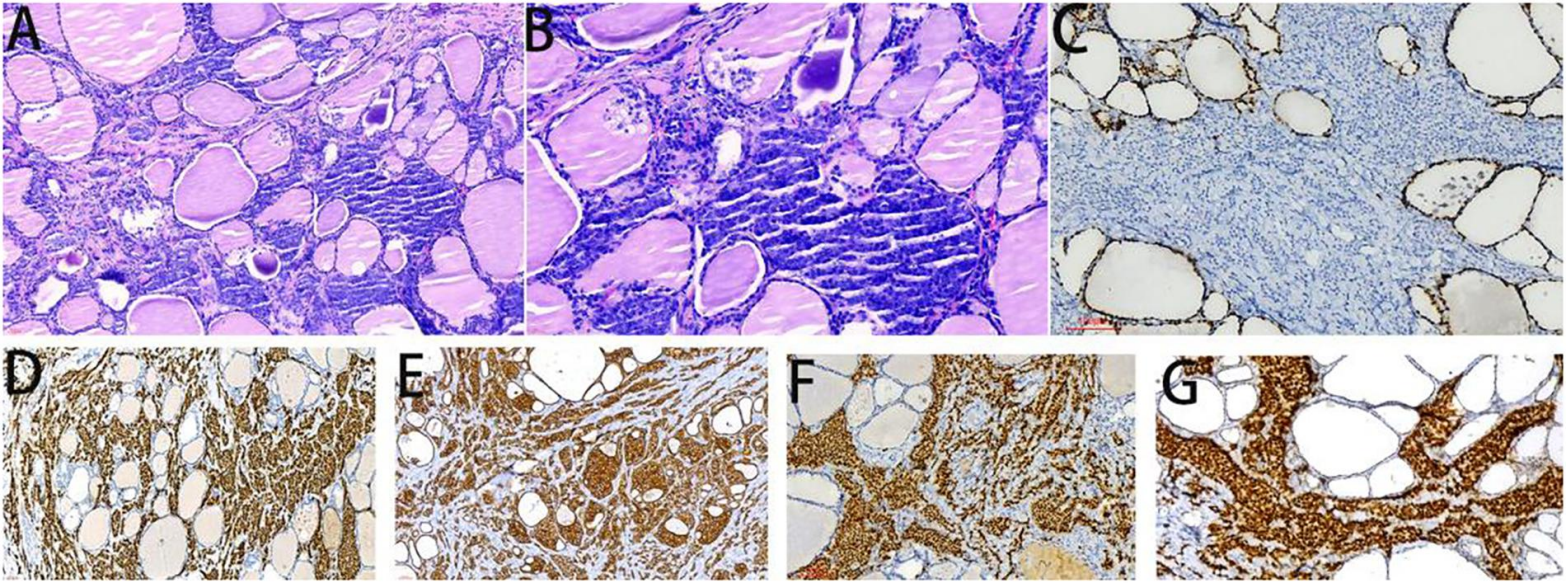
Histological and immunohistochemical examination results [**(A)** HE × 200, **(B)** HE × 400, **(C)** TTF-1 × 100, **(D)** ER × 200, **(E)** CK × 200 **(F)** GATA-3 × 100, **(G)** TRPS-1 × 100].

**Figure 4:**
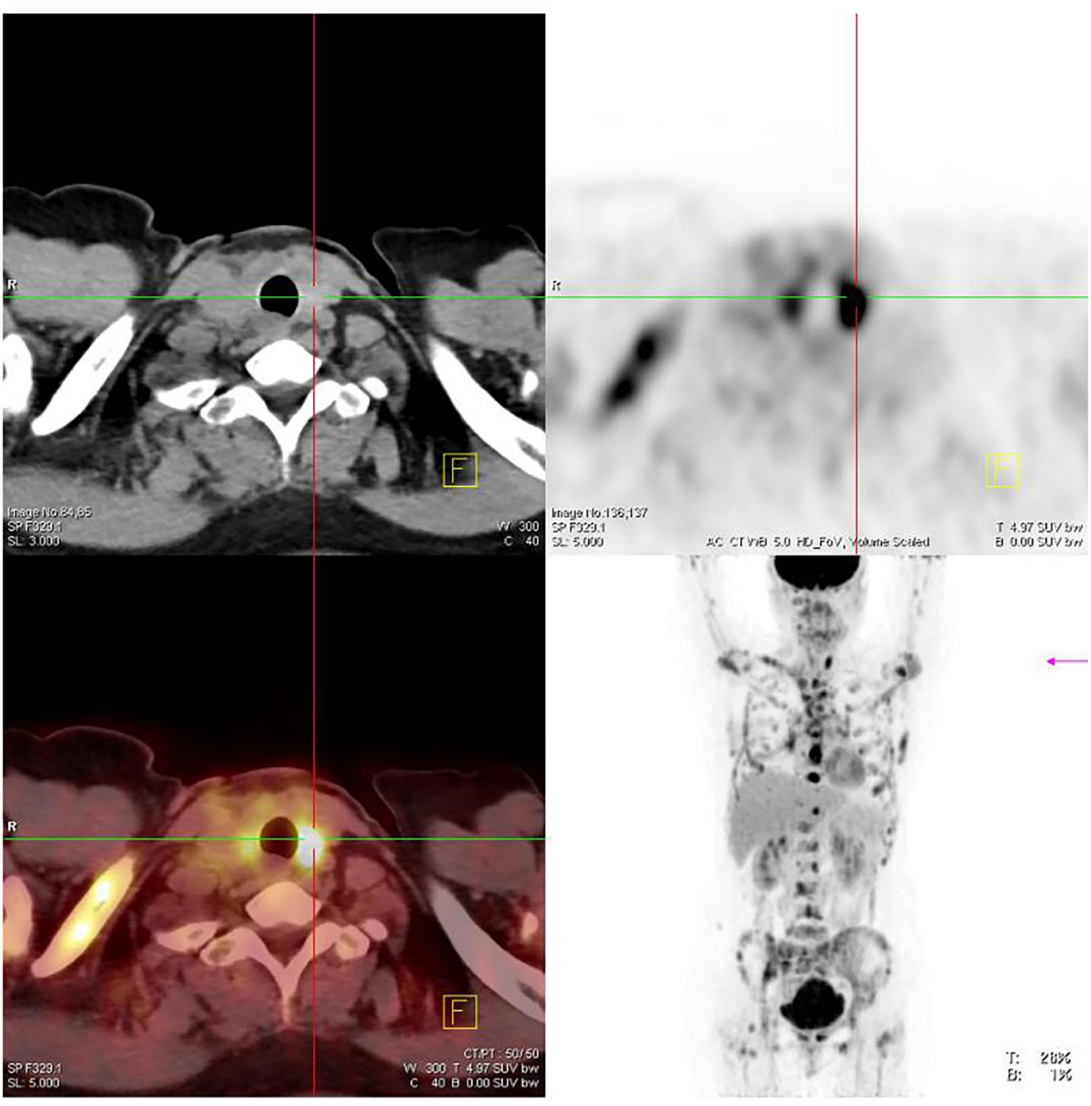
PET-CT indicated multiple systemic bone metastases.

A 54-year-old woman underwent radical left mastectomy 16 years prior to presentation to our hospital. Postoperative pathological examination revealed the diagnosis of acute invasive ductal carcinoma + invasive lobular carcinoma, metastases in 6/18 lymph nodes (T2N2M0, stage IIIA). Immunohistochemical examination revealed ER(++), PR (++), HER2 score 1+, Ki-67 index: 20%. Subsequently, she received radiotherapy, chemotherapy for six cycles, and tamoxifen endocrine therapy for 5 years. In April 2023, the patient underwent physical examination. Multiple nodules were observed in the right lobe of the thyroid gland, the largest one approximately 0.74 × 1.05 cm in the middle, with low echo, unclear boundary, irregular shape, vertical position, with visible punctate strong echo and punctate blood flow (C-TIRADS 4B) (Figures 1A,B). Enhanced CT shows significant enlargement of the right lobe, with a circular low-density shadow in the middle and moderate enhancement. The following laboratory results were obtained: Thyroid peroxidase antibodies (TPOAb): 83.64 IU/mL (0.00 IU/mL-5.61 IU/mL). A hard mass of approximately 1 cm diameter was palpated in the right lobe of the thyroid and could move up and down with swallowing without tenderness; however, the patient demonstrated no symptoms, such as difficulty in breathing or swallowing, hoarseness in voice, coughing, or other uncomfortable symptoms. The imaging and laboratory results raised high suspicion of malignant thyroid tumor. We advised the patient to undergo fine-needle aspiration biopsy (FNAB) to obtain a definite diagnosis. However, because of personal reasons, the patient refused to undergo FNAB. Therefore, the definitive diagnosis primarily relied on postoperative pathology. A unilateral thyroidectomy was performed based on the treatment principles of thyroid cancer. Intraoperatively, the right thyroid lobe was firm with an ill-defined mass without encapsulation with a gray-white border and yellowish center and poor glandular activity (Figures 2A,B). Pathological report indicated cancer originating from the breast. Immunohistochemical examination revealed calcitonin(-), TTF-1(-), Tg(-), CK(+), ER(90%+), HER2 score 2+, GATA-3(+), Ki-67 index: 20%, PR(40%+), and TRPS1(+), E-cadherin membranous positive (+) (Figures 3A–G). Positron emission tomography–computed tomography (PET-CT) showed multiple bone, mediastinal, and hilar lymph node metastases (Figure 4). Results of fish gene detection were negative. After multidisciplinary consultations of oncology, breast surgery, thyroid surgery, and chemotherapy, adjuvant chemotherapy for six courses and combined with endocrine therapy was administered, the timeline of the disease progression was shown in Supplementary Figure 1. Currently, the patient is alive and healthy, and follow-up checks have revealed no complications.

## Discussion

### Epidemiology of TMC

The incidence of thyroid cancer is increasing (3). Willis et al. suggested that as the thyroid has an abundant blood supply (approximately 560 mL/min in 100 g tissue) and fast blood flow, which is not conducive to tumor cell adhesion. High-iodine and high-oxygen environments are not conducive to tumor growth (4). Therefore, thyroid metastatic cancer is rare in clinical practice and accounts for only 1.25%–24% in autopsies (5). However, changes in the cell environment, such as insufficient or interrupted blood supply (6) or thyroid diseases (such as thyroiditis or nodular goiter) may increase the chance of tumor adhesion. This patient's TPOAb reached a level of 83.64 IU/mL, which is 14 times higher than the normal value, indicating a risk factor for thyroid metastasis.

### The diagnosis of TMC

TMC has no specific clinical symptoms and characteristics and is often discovered during physical examination (7). Only a few cases present with difficulty in breathing or swallowing because of the tumor being adjacent to the trachea or esophagus (8). Ultrasound is preferred for thyroid disease screening. TMC can be characterized by hypoechoic nodules with unclear boundaries and angiogenesis within the nodules (9). Saito et al. classified TMC into diffuse and nodular. The diffuse TMC is mainly characterized by diffuse hypoechoic changes throughout the thyroid, while the nodular TMC is characterized by hypoechoic nodular lesions with low-grade vascularization (10). However, the above are not specific changes in TMC; therefore, it does not offer the advantage of distinguishing primary thyroid diseases from secondary thyroid tumors. In such cases, FNAB is of great value (11). The accuracy of FNAB in diagnosing thyroid malignant tumors is as high as 87% and in TMC is as high as 93% (12), particularly in cases of breast cancer (13). Owens et al. demonstrated that the cytological characteristics of TMC are enlargement of the nucleus, irregular cell contour, and absence of nuclear invagination and pseudo inclusion bodies (14). However, Mager et al. suggested that the morphological features of this tumor are sometimes difficult to distinguish (15) from C-cell proliferation and medullary thyroid carcinoma (16). Therefore, immunohistochemistry plays an important role (17). TTF-1, Tg, calcitonin, and PAX8 are specific to primary thyroid cancer (18, 19). GATA-3 (20) mediates the heredity and differentiation of multiple cells and is a sensitive marker of breast cancer. MGB (21) is closely related to the growth of breast epithelium. ER, PR, HER-2, and GCDFP15 are also specifically expressed in breast cancer (5). Based on immunohistochemical results in our case, we could confirm that the tumor originated from the breast.

### Treatment of TMC

At present, there is no unified treatment guideline for TMC, and the treatment plan needs to be determined comprehensively based on the location of the primary tumor, pathological type, number of metastases, and the patient's physical state and willingness. Currently, surgical treatment is still the most commonly used treatment (22). Although metastasis to thyroid belongs to distant metastasis, surgical treatment can still achieve cancer control or even long-term cure when the thyroid is the only metastatic lesion (23). For unilateral thyroid metastasis, thyroid lobectomy is sufficient to resect the tumor, reduce tumor burden, and avoid compression symptoms. This approach can also preserve thyroid function, reduce the risk of recurrent laryngeal nerve injury and hypoparathyroidism, and improve long-term quality of life (9). For patients with bilateral, multifocal, or diffuse metastasis, total thyroidectomy is more appropriate to achieve local control (24). Cervical lymph node metastasis is not common, preventive neck lymph node dissection is not necessary in order to reduce postoperative complications (25). The presence of multiple metastases throughout the body often indicates a poor prognosis. According to the NCCN, a combination of chemotherapy or endocrine therapy should be administered to improve survival (26). However, when the tumor is large or associated with compression symptoms, such as difficulty in breathing and swallowing, local palliative surgical resection can improve the patient's living conditions and enhance their quality of life (8). TMC is not sensitive to radioactive iodine, therefore I131 treatment is not the preferred option (26). Radiation therapy can be considered for symptomatic relief in individuals at high anesthesia risk or those who are intolerant to surgery (17). Therefore, appropriate surgery combined with systemic therapy can help improve survival and prognosis, even in the setting of distant metastasis.

Our patient presented only with a metastatic lesion in the right lobe of the thyroid (9). Considering the protection of the parathyroid glands and recurrent laryngeal nerves, as well as the reduction of postoperative complications, thyroidectomy was performed. Although FNAB was not performed before surgery and multiple systemic metastases by PET-CT were found after surgery, the treatment was appropriate. As thyroid is adjacent to the trachea and esophagus, active thyroidectomy reduced tumor burden, avoided subsequent tumor compression symptoms and improved the patient's quality of life. If the FNAB and PET-CT were performed before the operation, the tumor nature and multiple systemic metastases could have been confirmed before the surgery. A multi-disciplinary consultation should be conducted before surgery and collaborative decision making should be undertaken to ensure better treatment outcomes.

### The prognosis of TMC

Owing to the low incidence of TMC and lack of specific symptoms and characteristics, it is prone to misdiagnosis and underdiagnosis. Therefore, its prognosis is poor and the average survival time after treatment is only 43.2 months (2). Furthermore, its prognosis is related to the tumor type; number, size, and location of metastases; and the interval from the diagnosis of the primary tumor to metastasis (26). However, when thyroid metastasis is detected, approximately 35%–80% patients present with multiple-organ metastases; therefore, even with positive treatment, achieving a favorable prognosis is challenging (25). Thus, early diagnosis and treatment are crucial.

### Limitations and strengths

One limitation of this case is that we did not obtain definite pathological results from FNAB or radiological diagnosis from PET-CT before operation. However, because of our vast experience, we provided the patient with timely supplementary examination and treatment; currently, the patient is doing well. We believe that the diagnosis and treatment process of this case will provide guidelines for other clinicians who may encounter similar cases in clinical practice.

## Conclusion

TMC originating from the breast is a rare occurrence. If a patient has a history of breast cancer, the presence of newly formed thyroid nodules accompanied by thyroid dysfunction should raise high suspicion regarding tumor origin. FNAB before the operation is of high value in determining tumor nature and origin. Postoperative immunohistochemistry can identify tumor origin based on specific tumor markers. Surgical intervention is the preferred treatment, and the extent of thyroidectomy should be determined by considering factors, such as tumor location, number of metastases, patient life expectancy, and the nature of the primary tumor. Chemotherapy and endocrine therapy are also crucial, and collaboration is necessary to improve treatment outcomes.

## Data Availability

The raw data supporting the conclusions of this article will be made available by the authors, without undue reservation.

## Supplementary material

SUPPLEMENTARY FIGURE 1 The timeline of disease progression.
