# Supplementary figures and images for: Diagnosis and treatment of breast cancer metastasis to thyroid: a case report and literature review
Source: Front Surg. 2026 May 25;13:1787683. doi: 10.3389/fsurg.2026.1787683 (PMC13243277; doi:10.3389/fsurg.2026.1787683)

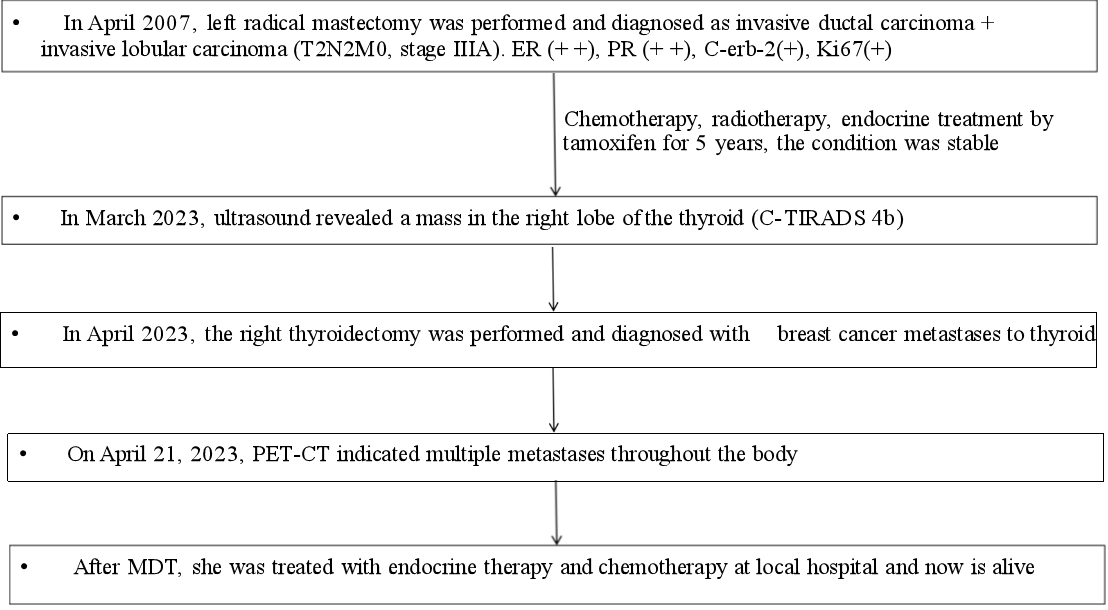

Supplement: SUPPLEMENTARY FIGURE 1 — The timeline of disease progression. [file Image1.jpeg]
